# Strategies to Improve Vaccine Efficacy against Tuberculosis by Targeting Innate Immunity

**DOI:** 10.3389/fimmu.2017.01755

**Published:** 2017-12-11

**Authors:** Ulrich E. Schaible, Lara Linnemann, Natalja Redinger, Emmanuel C. Patin, Tobias Dallenga

**Affiliations:** ^1^Cellular Microbiology, Priority Program Infections, Research Center Borstel, Borstel, Germany; ^2^Thematic Translation Unit Tuberculosis, German Center for Infection Research, Research Center Borstel, Borstel, Germany; ^3^Retroviral Immunology, The Francis Crick Institute, London, United Kingdom

**Keywords:** tuberculosis, vaccine, *Mycobacterium tuberculosis*, host-directed therapy, neutrophils, IL-10, CD1, cell death

## Abstract

The global tuberculosis epidemic is the most common cause of death after infectious disease worldwide. Increasing numbers of infections with multi- and extensively drug-resistant variants of the *Mycobacterium tuberculosis* complex, resistant even to newly discovered and last resort antibiotics, highlight the urgent need for an efficient vaccine. The protective efficacy to pulmonary tuberculosis in adults of the only currently available vaccine, *M. bovis* BCG, is unsatisfactory and geographically diverse. More importantly, recent clinical studies on new vaccine candidates did not prove to be better than BCG, yet. Here, we propose and discuss novel strategies to improve efficacy of existing anti-tuberculosis vaccines. Modulation of innate immune responses upon vaccination already provided promising results in animal models of tuberculosis. For instance, neutrophils have been shown to influence vaccine efficacy, both, positively and negatively, and stimulate specific antibody secretion. Modulating immune regulatory properties after vaccination such as induction of different types of innate immune cell death, myeloid-derived suppressor or regulatory T cells, production of anti-inflammatory cytokines such as IL-10 may have beneficial effects on protection efficacy. Incorporation of lipid antigens presented *via* CD1 molecules to T cells have been discussed as a way to enhance vaccine efficacy. Finally, concepts of dendritic cell-based immunotherapies or training the innate immune memory may be exploitable for future vaccination strategies against tuberculosis. In this review, we put a spotlight on host immune networks as potential targets to boost protection by old and new tuberculosis vaccines.

## Introduction

With 1.8 million deaths worldwide, the World Health Organization listed tuberculosis among the top 10 causes of death in 2015 (1) and positioned tuberculosis to be the number one killer after infectious disease. Alarmingly high numbers of cases with multidrug- (MDR) and extensively drug-resistant (XDR) variants, prompted the G20 leaders to single out tuberculosis within the emerging problem of antibiotic resistance in their 2017 summit declaration. Nearly, half a million people were identified to be infected with MDR strains of *Mycobacterium tuberculosis* in 2016 but not even half of them were treated successfully (1). A number of XDR tuberculosis cases are even considered untreatable. Those patients have a survival rate of only 30% (1). These figures highlight the global tuberculosis health crisis and emphasize how urgently novel vaccines are needed. As of today, the widely used attenuated live vaccine *M. bovis* BCG provides only limited protection. It is effective against primary tuberculosis during childhood, which can lead to severe outcomes, including meningitis. However, the protective efficacy of BCG against pulmonary tuberculosis in adults is unsatisfactory and varies tremendously between geographical areas, concretely the greater the distance from the equator the higher the efficacy (2). Consequently, huge scientific and financial efforts have been made to design and develop novel vaccine types, hoping to enhance protective immunity against tuberculosis in adults. The aims are either to sterilely eliminate the mycobacteria or—at least—prevent forms of active disease, such as contagious pulmonary tuberculosis in order to limit transmission. Still, none of the novel vaccine candidates have replaced BCG. A booster vaccine candidate with just one mycobacterial antigen, MVA85A, which was promising in animal models, failed to enhance BCG-primed protection in a recent clinical study in South Africa (3). In this approach, the secreted mycobacterial mycosyl transferase Ag85A, involved in the synthesis of trehalose dimycolate, cell wall maintenance, and *M. tuberculosis* survival (4), was cloned into a recombinant strain of modified Vaccinia Ankara virus (5) to be used as booster vaccine following BCG priming. Despite induction of antigen-specific multifunctional Th1 and Th17 cells in infants that received MVA85A on top of BCG priming (3), 1,399 vaccinees were not better protected from tuberculosis than the placebo controls, raising the question, whether BCG-centric vaccine strategies that aim to elicit potent Th1 cell responses against dominant antigens are still the most promising approaches (6).

A different approach to improve vaccine efficacy is to modulate host immune networks concomitantly or upon vaccination, using old and new vaccines to boost protection. Immune system networks may be exploited to bias immune responses toward protective immunity against tuberculosis. Various host responses following vaccination have been described to interfere with establishment of protective immunity similar as it is seen after natural infection (7). In this review, we discuss novel approaches, which may improve anti-tuberculosis vaccination. These include targeting neutrophils as well as the type of phagocyte cell death, i.e., necroptosis vs. apoptosis during vaccination. We review CD1-binding lipids as antagonists for CD1-restricted T cell responses, which may interfere with proper vaccine-mediated immunity. Additionally, immunoregulatory cytokines such as IL-10 may also affect vaccine efficacy and, thus, are putative targets for *vaccination-accompanying immunomodulation*. Finally, based on promising results in immunotherapy of cancer, we will consider whether induction of immunogenic cell death has the potential to enhance T cell responses.

## Neutrophils upon Vaccination: The Good, the Bad, or the Neutral for Vaccine Efficacy

**Figure 1:**
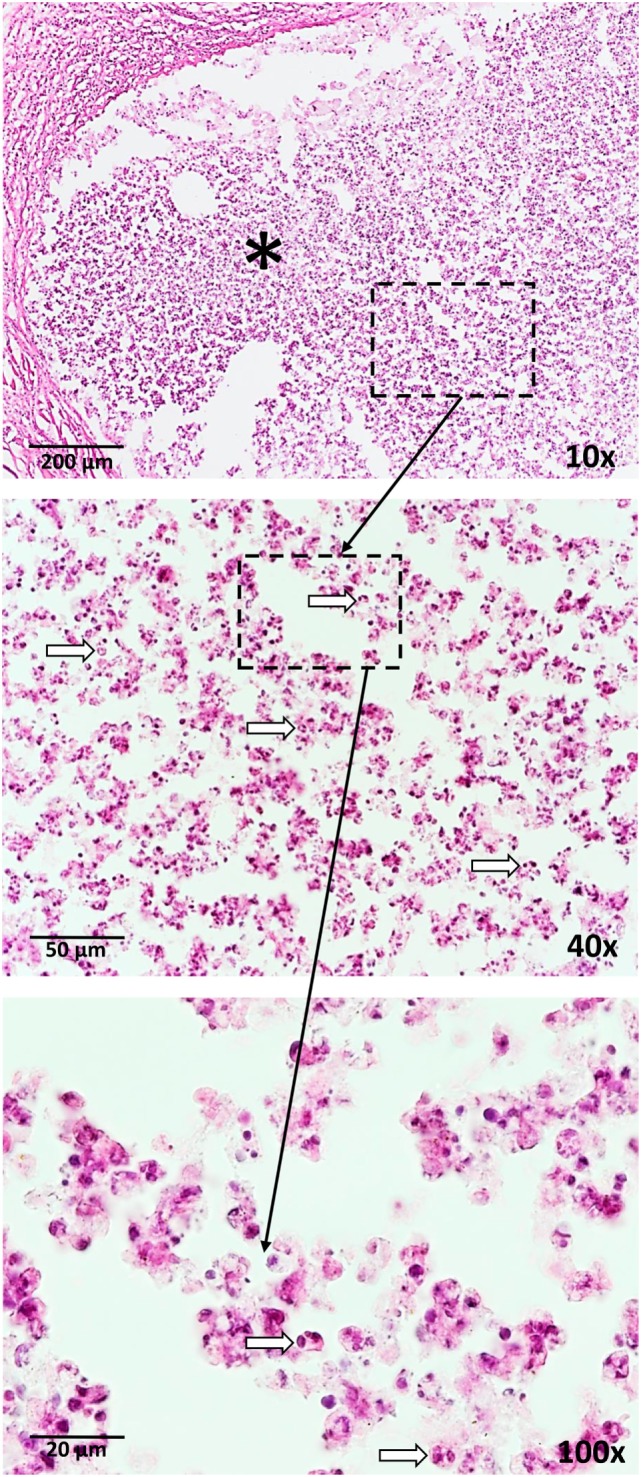
Neutrophil influx (arrows) is a response to vaccination with BCG as well as a trehalose-6,6-dibehenate (TDB)-containing liposome-based adjuvant. The photographs are derived from skin tissue of mice, which were subcutaneously vaccinated with TDB liposomes containing ovalbumin at the tail base. Thirty-one days after vaccination, cryosections were stained with hematoxylin and eosin. * = injection site.

Neutrophils are associated with disease in patients with active tuberculosis and susceptible mouse strains developing necrotic granulomas similar to humans. These potent anti-microbial innate immune cells have been shown to represent the main cell population in bronchoalveolar lavage and sputum of patients with active pulmonary tuberculosis and they carry the main mycobacterial load (8). Berry et al. identified a blood mRNA profile, which was dominated by a neutrophil signature and allowed to discriminate between active tuberculosis patients, latently infected ones, and healthy controls (9). Moreover, neutrophil-rich lesions associated with necrotic caseous material were found in resected lungs of patients (10). Thus, active pulmonary tuberculosis is considered to be neutrophil-driven (11). Susceptible mouse models, mimicking the pathology of human tuberculosis, such as C3HeB/FeJ, DBA/2, and I/St, show strong contribution of neutrophils to mycobacterial load, tissue destruction, pathology, and decreased survival rates (12–14). Importantly, not only in active *M. tuberculosis* infection but also after subcutaneous BCG vaccination, neutrophils rapidly enter the site of injection in large numbers (15). Neutrophil influx is also observed in response to vaccination using the synthetic trehalose dimycolate analog, trehalose-6,6-dibehenate (TDB), in a liposome formulation as adjuvant at the site of subcutaneous injection (Figure 1). Trehalose dibehenate was proposed as adjuvant to boost efficacy of subunit vaccines against tuberculosis (16). Neutrophils have also been shown to shuttle bacterial antigens to draining lymph nodes for T cell priming (17). However, contradictory roles for neutrophils regarding vaccine efficacy have been described.

Neutrophils have been shown to activate B cells leading to antibody secretion. After reprogramming by IL-10, among other signals, neutrophils entered the marginal zone of the spleen upon mucosal colonization by microbes in murine neonates (18). Here, the neutrophils exhibited a B cell–helper phenotype and induced immunoglobulin diversification and production by T cell-independent B cell activation. However, splenic B cell–helper neutrophils have not been detected in humans so far (19). Nagelkerke et al. identified a homogenous neutrophil population in human spleen that did not exhibit enhanced capabilities over blood neutrophils regarding co-stimulation of B cells and antibody production thereof. Classically, B cell-mediated immunity was thought to contribute only little to protection against tuberculosis. However, more recent studies reveal the potential of specific antibodies to contribute to immune protection against *M. tuberculosis* infection as comprehensively reviewed elsewhere (20, 21). In another study, murine bone marrow-derived neutrophils have been shown to exhibit a neutrophil–dendritic cell hybrid phenotype when cultured with GM-CSF (22). Those cells showed both, typical neutrophil properties, like NET formation and cathelicidin-mediated bacterial killing as well as intrinsic abilities of dendritic cells, like expression of MHC class II and co-stimulatory molecules and IL-12 production. The cells were capable of antigen presentation to T cells. Therefore, shifting the neutrophil phenotype elicited by vaccination toward this unusual subtype, for example, by concomitant administration of rGM-CSF, may strengthen vaccine efficacy. This subtype of antigen-presenting neutrophil, named N_β_, was identified to migrate to the site of HIV infection in an NF-kB-dependent manner and activate specific CD8^+^ T cells (23). However, the function of N_β_ neutrophils in tuberculosis awaits further analysis. After experimental administration of the recombinant *M. smegmatis*-based vaccine mc^2^-CMX, which expresses fragments of the *M. tuberculosis* antigens Ag85c, MPT51, and HspX, and elicits specific Th1 and Th17 cells, neutrophil-rich lesions with a necrotic centers were observed (24). When neutrophils were depleted during vaccination, Th1- and Th17-specific responses, responsible for reduction of mycobacterial loads were nullified as were the protective efficacy of the mc^2^-CMX vaccine.

In contrast to immunity-promoting functions of neutrophils, effects detrimental for cellular and humoral immunity have also been described. Neutrophils can interfere with cell-mediated immunity upon adjuvant administration (25). Neutrophil depletion for the first 24 h after immunization restored CD4^+^ T and B cell responses and improved clustering of antigen-specific T cells with DCs, as shown by intravital microscopy. The strong suppressive effect of neutrophils on T and B cell activation depended on the small lipid mediators prostanoids since neutrophil influx into the lymph nodes was abrogated in cyclooxygenase (COX)-1 and -2 knock-out mice as well as when specific inhibitors for these two enzymes were applied (26). Inhibitors included COX-1-selective SC-560, COX-2-selective NS-398, and Indomethacin. Specifically, the first wave of rapid neutrophil entry was dependent on prostaglandins, while the authors identified the neutrophil-derived eicosanoid thromboxane A_2_ to control the extent of T cell responses. Indeed, thromboxane A_2_ has been shown to negatively regulate physical contact of DCs and T cells. Treatment by an exogenous thromboxane receptor agonist led to random, not chemokine gradient-guided T cell movement. Thromboxane receptor-deficient mice showed increased immune responses to a model antigen (27).

Taken together, these data demonstrate that neutrophil influxes and cross-talk between neutrophils and professional antigen-presenting cells can negatively influence T cell priming and proliferation upon vaccination. Importantly, neutrophil depletion or neutralization of neutrophil-derived immune mediators restored protective T cell responses upon adjuvants administration. Thus, targeting neutrophil-mediated regulation of the dendritic cell—T cell synapse may improve the establishment of adaptive immunity. Moreover, the neutrophils’ short life span of only a few days suggests the type of cell death, e.g., immuno-silent apoptosis vs. immunogenic apoptosis vs. necrotic cell death, as an additional target during immune priming.

## Impact of the Type of Immune Cell Death on Vaccine Efficacy

For a long time, apoptotic cell death has been thought to be immuno-silent or even anti-inflammatory. This paradigm shifted when it has been shown that the trigger of apoptosis shapes the immunological consequences thereof. For instance, Fas ligand-mediated apoptosis, e.g., of tumor cells, induces strong inflammatory responses through inflammasome activation, i.e., caspase-1 and its IL-1β- and IL-18-activating properties (28). Importantly, mycobacteria-infected macrophages succumbing to apoptosis deliver foreign antigens to dendritic cells and subsequently cross-prime antigen-specific T cells, thereby, establishing protection against experimental tuberculosis in mice (29, 30). Dendritic cells were activated by mycobacterial pathogen-associated molecular patterns carried along by apoptotic vesicles from infected cells. In contrast to virulent *M. tuberculosis*, BCG induces apoptosis in macrophages, especially upon IFNγ activation (31). These findings are corroborated by us as well as others showing that attenuated *M. tuberculosis* strains as well as BCG primarily induce apoptosis in neutrophils, whereas virulent *M. tuberculosis* strains induce necrotic cell death in both, macrophages and neutrophils (11, 32–34). Moreover, apoptosis has been described as a defense mechanism against *M. tuberculosis* (35). In contrast, Aporta et al. report that BCG as well as the live attenuated vaccine candidate *M. tuberculosis* strain SO2, which lacks phoP, does not induce cell death (apoptosis or necrosis) in murine bone marrow-derived macrophages and the murine macrophage cell line J774, while the virulent parental clinical isolate MT103 induces apoptosis, but not necrosis (36). However, the contradicting findings regarding macrophage cell death induced by *M. tuberculosis* infection, i.e., necrosis vs. apoptosis, may be associated with the different mycobacterial strains (e.g., MT103, H37Rv, and Erdmann) or the macrophage types used (e.g., murine bone-marrow-derived, human monocyte-derived, murine, or human macrophage cell lines), respectively (37–39). Of note, the novel anti-tuberculosis vaccine candidate *M. bovis* BCG VPM that expresses listeriolysin but is deficient in urease, is a better inducer of macrophage apoptosis than the parental BCG strain (40).

The efficacy of whole cell anti-cancer vaccines has been shown to depend on the type of induced cell death prior to administration. In a prophylactic cancer vaccination study, apoptotic vs. necrotic tumor cells were studied for their efficacy to induce tumor antigen-specific T cells (41). Vaccination with γ-irradiated apoptotic tumor cells prevented tumor outgrowth in up to 100% of the mice. In contrast, mice that received necrotic tumor cells, generated by three freeze-thaw cycles, were protected in only 0–30% of the cases. The apoptotic tumor cell vaccine recruited predominantly dendritic cells as well as CD4^+^ and cytotoxic CD8^+^ T cells at the site of injection. Injection of necrotic tumor cells elicited strong macrophage influx. Apoptotic tumor cells increased the survival rates of mice. These data suggest that antigens delivered by apoptotic cells to antigen-presenting cells can result in a stronger T cell response than those carried by necrotic cells (42). This notion is corroborated by a study, which combined dendritic cell-based immunotherapy with immunogenic cell death to successfully elicit Th1-mediated immunity to glioma (43). Application of necroptotic tumor cells also proved to be a successful vaccine strategy against cancer in mice. Induction of necroptosis in tumor cells by triggering RIPK3 resulted in the release of damage-associated molecular pattern molecules, subsequent dendritic cell activation, and priming of tumor antigen-specific IFNγ-producing T cell (44). Therefore, necroptotic tumor vaccines may also be employed, which comes in handy since many tumors are resistant to apoptosis induction.

These data indicate that the type of immune cell death upon whole cell anti-tumor vaccination shapes elicited immune responses. However, vaccination strategies, which determine the type of cell death of the antigen-bearing cells, may also improve anti-microbial vaccines. Application of an influenza vaccine with a microneedle following non-ablative fractional laser pretreatment triggered death of antigen-presenting cells and dsDNA release. This activated the cGAS/STING pathway and led to protective immunity (45). The use of this technique for BCG vaccination was effective without generating obvious inflammatory lesions (46). Analysis of the composition of infiltrating immune cells and their fate after tuberculosis vaccine application may improve vaccines by locally applying cell death inhibitors or inducers at the vaccination site. Upon immunization, programed cell death associated with the release of DAMPs promotes Th1-mediated immunity, whereas unregulated necrosis fails to induce CD4^+^ T cell responses. Certain self-lipid antigens have been shown to antagonize activation of T and natural killer (NK) T cells (47). One might envisage that oxidized lipid antigens, potentially generated during host phagocyte necrosis, interferes with induction of immunity by vaccination as discussed below.

## Lipid Antigen Antagonists to Control Vaccination Efficacy?

The ability of lipid antigens to prime-specific T cells upon presentation by myeloid antigen-presenting cells *via* highly conserved CD1 molecules indicates the possibility of mycobacterial lipid-based vaccinations in an MHC-independent manner. Human antigen-presenting cells can express four CD1 isoforms (CD1a–d) that bind a variety of exogenous and endogenous lipid compounds (48–50). The relevance of CD1-restricted lipid antigens for T cell immunity has been well characterized for autoimmune diseases (51), cancer (52), and infections (53).

Human CD1b can bind and present mycobacterial antigens including mycolic acids and glucose monomycolate to T cells *in vitro* (54). Other CD1b ligands include mycobacterial diacylated sulfoglycolipids (Ac_2_SGL) and phosphatidyl-myo-inositol dimannosides (PIM_2_) (55). Furthermore, CD1^+^ dendritic cells have been isolated in great numbers from leprosy skin lesions upon *M. leprae* infection, which was associated with active cellular immune response (56). Importantly, mycobacterial lipid antigen-reactive T cells were identified in blood of patients with latent and active, antibiotic-treated tuberculosis (57).

Emphasizing lipid antigen involvement in mycobacterial immune responses, direct immunization of guinea pigs with a mixture of mycobacterial lipid antigens induced CD4-negative cytotoxic T cells reacting to CD1-expressing, mycobacterial antigen-presenting dendritic cells (58). Another study found that vaccination of guinea pigs with *M. tuberculosis* lipids-containing liposomes in combination with a non-lipid adjuvant, prior to *M. tuberculosis* infection, reduced pathology and mycobacterial loads, presumably due to CD1-restricted T cell responses (59). Similar protection was observed in guinea pigs by a vaccine combining Ac_2_SGL and PIM_2_ (60). Especially, Ac_2_SGL showed promising results, activating human CD1-restricted CD8^+^ T cells *in vitro* isolated from skin test-positive donors (55). However, long-term memory protection was not detected even though glycolipid-specific immunoglobulin responses can occur after lipid antigen presentation (61, 62). To induce memory B cell responses against lipids, well-established anti-protein vaccine formulations were combined with glycolipids by conjugating glucose monomycolate to a carrier protein (63). Disappointingly, anti-glucose monomycolate antibody responses were not induced.

Even though indications for protective effects upon immunization with mycobacterial lipid antigens were observed, detailed knowledge of the mechanisms, interaction characteristics, and downstream effects remain largely unknown. Most studies based on mycobacterial lipid vaccination, lack data on long-term memory responses, and prolonged immunity to tuberculosis.

Besides exogenous lipids, all CD1 molecules can bind and present a broad variety of endogenous, self-derived phospholipids, and glycosphingolipids with differential effects on subsequent T cell activation. Endogenous skin lipids presented by antigen-presenting cells *via* human CD1a molecules have been shown to either inhibit or stimulate T cell responses dependent on the structural properties and charges of the lipid antigen. In contrast to lipids with hydrophilic head groups that protrude into the binding groove of CD1 and, thus, prevent T cell receptor binding and subsequent T cell activation, highly apolar lipids placed deep in the binding groove, facilitated strong T cell receptor binding, and induced an autoreactive phenotype of CD1a-restricted T cells (48). Therefore, autoreactive T cells can become activated upon a shift toward a hydrophobic head group lipid composition, which may occur during certain autoimmune skin diseases (64, 65). Self-derived lipid antigens also indirectly influence the presentation of mycobacterial lipids and downstream signaling. Structural crystallography analysis revealed that CD1b adapts to different sizes and numbers of mycobacterial lipid alkyl chains by capturing small endogenous gangliosides, thus, stabilizing the CD1–lipid complex (66). Huang et al. identified deoxyceramides and diacylglycerols species as scaffolding lipids (47). They were found to nest deeply into the binding groove of CD1 molecules and, thereby, affect binding of exogenous mycobacterial lipid antigens. The efficacy of interference depended on the length of the hydrophobic side domains. Hydrophobic long alkyl side chains, protruding into the binding groove, inhibited T cell activation, whereas those with short hydrophobic domains stabilized the lipid–CD1 complex and facilitated T cell activation (47). These studies strongly suggest that the immune system is able to sense inflammation-induced changes of the lipidome within affected tissues, which in turn modulates immune responses. Inhibition of CD1–T cell receptor interaction by protruding lipids interrupts T cell activation. Therefore, self-derived lipids, serving either as scaffolding molecules or as auto-antigens, may function as agonists or antagonists for downstream T cells activation depending on their molecular structure. Recent studies tried to utilize these findings for development of new therapeutics. CD1d-restricted invariant NK T cells were strongly activated to produce Th1 and Th2 cytokines by recognition of a CD1d–α-galactosylceramide complex (67) indicating α-galactosylceramide as a putative adjuvant for vaccination. Addition of a phenyl group or a shorter phytosphingosine chain, as in the analog OCH, changes CD1d–antigen complexes on antigen-presenting cells and shifts downstream NKT cell responses to either IFNγ or IL-4 production, respectively (67). These modifications can shape immune responses including prevention of autoimmunity, such as experimental autoimmune encephalomyelitis in mice (68) and improved cancer therapy by induction of NKT cell-driven Th1 responses (69, 70) suggesting that CD1–lipid–T cell receptor interactions can be targeted to improve vaccine efficacy.

Upon release of reactive oxygen species, human neutrophils oxidized self-lipids by their own myeloperoxidase (71, 72). Initial oxidation by a single free radical could trigger an oxidization cascade, which proceeds within biological membranes, resulting in accumulation of oxidized lipid species by just one hit (73). Importantly, we have shown that human neutrophils underwent necrotic cell death upon infection with *M. tuberculosis* that was dependent on myeloperoxidase-generated reactive oxygen species (32). Therefore, infected necrotic neutrophils, which are removed by myeloid cells capable to present antigens *via* CD1 molecules, represent a source of oxidized self-lipids that potentially interfere with T cell responses to mycobacteria. Exogenous and endogenous lipid antigens are interesting compounds to control CD1 antigen presentation and, thereby, silencing or activating CD1-restricted T cell responses in order to modulate immunity against mycobacteria during infection or vaccination with whole attenuated mycobacteria. Interestingly, CD1-restricted *M. tuberculosis*—as well as pollen lipid-specific T cells have been shown to produce the anti-inflammatory cytokine IL-10 (74, 75), which is able to interfere with BCG vaccination efficacy (76, 77).

## Neutralizing Anti-Inflammatory IL-10

Immune responses during infection have to be tightly controlled in order to avoid excessive inflammation and subsequent tissue damages. Anti-inflammatory cytokines such as IL-10 prevent immunopathology by restraining both, Th1 and Th17 cell activities (78). Due to its negative effect on protective adaptive immune responses, IL-10 signaling has been associated with detrimental course of infection in experimental tuberculosis in mice (77, 79). Importantly, increased IL-10 production upon vaccination against various pathogens was shown to limit their protective efficacy (80, 81). Several studies reported induction of IL-10 upon BCG challenge in both, humans and mice (82–85). IL-10-deficient mice vaccinated with BCG revealed higher splenocyte numbers generating IFN-γ, IL-17A, and TNF-α when compared with wild-type ones (76, 77). Accordingly, BCG-vaccinated mice were better protected against *M. tuberculosis* infection after treatment with an IL-10-neutralizing antibody (76, 77). Thus, controlled short-term regulation of IL-10 levels during BCG vaccination represents a promising host-directed immune modulation to expedite mycobacterial clearance, establish better T cell-mediated immunity and thereby improve vaccination efficacy.

Agonists for C-type lectin receptors, particularly trehalose dibehenate (TDB), have been highlighted as promising adjuvant candidates for anti-tuberculosis vaccination (86). TDB is the synthetic analog of the mycobacterial cell wall glycolipid trehalose-6,6′-dimycolate (TDM), which binds to the macrophage-inducible C-type lectin, also known as Mincle, and subsequently induce pro-inflammatory cytokine secretion (16, 87, 88). Interestingly, Lindenstrom et al. demonstrated that CAF01, a liposome preparation containing TDB, could trigger potent and sustained Th1 and Th17 responses (86). Mincle was identified as target for vaccine adjuvants with strong immunogenic properties against *M. tuberculosis* infection in mice (89). We recently showed that Mincle signaling was also critical for IL-10 secretion in response to TDM or BCG challenge *in vitro* (90). Our studies demonstrated that IL-10 secretion down-regulated IL-12 production, a pivotal cytokine for proper induction of Th1 responses (91). Thus, it would be interesting to investigate whether anti-tuberculosis vaccines including those formulated with TDB or other Mincle ligands can be combined with anti-IL-10 antibodies to improve both, efficacy and duration of protection against *M. tuberculosis*. It remains to be elucidated whether this approach can be extended to other anti-inflammatory cytokines such as TGF-β and IL-27. In conclusion, controlled short-term down-regulation of IL-10 levels after vaccination may be a promising host-directed modulation to drive a Th1-/Th17-mediated memory response and promote long-term immunity. In this context, regulation of immune cell populations that interfere with developing Th1-derived protection upon vaccination, e.g., myeloid-derived suppressor cells (MDSC) and regulatory T cells (T_regs_), have the potential as pharmaceutically controllable switch points to improve long-term vaccine efficacy.

## Controlling Th1-Supressing Immune Cell Subpopulations

Depletion of immune cell subsets, such as MDSC and T_regs_ by monoclonal antibodies have been shown to improve clinical outcome and vaccination efficacy (92). Increased frequencies of MDSC have been shown in blood samples and at the site of *M. tuberculosis* infection in chronic as well as recently infected patients (93–95). MDSC suppressed CD4^+^ and CD8^+^ T cell proliferation and cytokine secretion *ex vivo*. After successful anti-tuberculosis treatment, MDSC frequencies became normalized accompanied by MDSC maturation as indicated by expression of co-stimulatory surface molecule CD80. Importantly, when MDSC were depleted in *M. tuberculosis*-infected mice by administration of all-*trans* retinoic acid, mycobacterial burden, and pathology in lungs were reduced (96). In tumor-bearing mice, researchers found that MDSC migrate along a gradient of the pro-inflammatory alarmin S100A8/9 leading to MDSC accumulation (97). Blockade of S100A8/9 binding to MSDC resulted in decreased frequencies of MDSC. Moreover, mice deficient for S100A9 generated potent tumor rejection responses, while S100A9 overexpression led to accumulation of MDSC and reduced numbers of differentiated dendritic cells and macrophages. Of note, S100A8/9 comprise 45% of all cytosolic proteins in neutrophils, while its amount is 40× lower in monocytes (98). As mentioned above, massive infiltration of neutrophils at the site of BCG vaccination as well as *M. tuberculosis* infection may be the reason for the reported upregulated levels of S100A8/9 during tuberculosis (99, 100), which may result in accumulation of MDSC and, ultimately, suppression of proper Th1 immune responses. Indeed, S100A8/9, specifically derived from neutrophils, was responsible for diabetes-induced thrombocytosis (101). Blockade of S100A8/9 binding to its receptor by administration of Paquinimod was beneficial. Specific inhibition of S100A8/9 by Paquinimod reduced inflammation and pathology under various disease conditions, including leukocyte recruitment during sterile inflammation, experimental systemic sclerosis, and experimental osteoarthritis (102–105). Therefore, MDSC may represent the S100A8/9-driven connection that links massive neutrophil influx to impaired Th1 immune responses and memory phenotypes thereof. Vaccination efficacy may benefit from treatments regulating MDSC influx or their maturation during vaccination-induced immune priming.

Another immune cell subpopulation playing a detrimental role in the successful establishment of Th1-mediated control of *M. tuberculosis* infection are T_regs_. Increased frequencies of FoxP3^+^, IL-10-, and TGFβ-releasing T_regs_ have been found in peripheral blood and at the site of infection in tuberculosis patients (106–108). After successful treatment, those frequencies decreased to similar levels as in healthy controls (108). For mice, it has been shown that T_regs_ are responsible for impaired generation of protective immunity against tuberculosis. After aerosol infection of mice, preexisting *M. tuberculosis*-specific T_regs_ expanded in the draining lymph nodes (109). Accumulation of those T_regs_ in the lungs resulted in delayed arrival of pathogen-specific IFNγ-producing CD4^+^ and CD8^+^ effector T cells and suppression of protective immunity. Induced expansion of both, T_regs_ and IFNγ-, perforin-producing T effector cells by s.c. administration of rIL-2 led to accumulation of those subsets in the lungs of *M. tuberculosis*-infected macaques (110). Interestingly, infiltrating Th1 effector cells and T_regs_ orchestrated both, control of *M. tuberculosis* burdens as well as resistance to severe inflammation and tissue damage. Targeted inhibition of Th2 and T_regs_ generation by administration of the small compounds suplatast tosylate and D4476 reduced *M. tuberculosis* burden and established a protective Th1 immune response (111). The same research group shows in a follow-up study that the same treatment enhanced BCG efficacy upon vaccination so that *M. tuberculosis* burdens were reduced in lungs and spleens of mice after infection (112). Mycobacteria-specific T_regs_ already occur after administration of commonly used BCG formulations as well as during clinical trials with novel candidate vaccines (113). To enhance vaccine efficacy and Th1-mediated immunity upon BCG vaccination against *M. tuberculosis* infection, T_regs_ have been successfully targeted. Dhiman et al. showed that human NK1.1^+^ NK cells promoted CD8^+^ T cell responses and concomitantly reduced frequencies of *M. tuberculosis*-specific T_regs_ in an IL-22-dependent manner (114). BCG vaccination of mice generated those IL-22- and IFNγ- secreting NK cells and their specific depletion upon vaccination led to higher numbers of T_regs_, increased mycobacterial burden, and reduced T cell responses after *M. tuberculosis* infection. More importantly, concomitant administration of rIL-22 and BCG in NK1.1^+^ cell-depleted animals restored CD4^+^ T cell responses and BCG vaccination efficacy, indicating that IL-22 treatment can improve vaccination efficacy. Another study shows that a novel subunit vaccine booster candidate induced strong and specific Th1-mediated IFNγ and IL-2 production, while T_regs_ were down-regulated (115). This formulation included a fusion protein of Ag85B, Mpt64, and Mtb8.4 formulated in an adjuvant containing dimo-thylidioctyl ammonium bromide and BCG-polysaccharide-nucleic acid, consisting of polysaccharides and nucleic acids extracted from BCG. Finally, subsequent administration of a T_regs_-depleting anti-CD25 antibody upon BCG vaccination, resulted in an increased IFNγ response, and reduced mycobacterial load upon *M. tuberculosis* infection (116).

Thus, temporal regulation of highly relevant immune subpopulations, such as MDSC and T_regs_ that otherwise modulate immune responses and protect from exacerbated inflammatory tissue destruction, may display a crucial step in shifting the immune memory toward a protective Th1 response upon vaccination with BCG-based vaccines. Besides other T cell populations, which also modulate immune responses upon vaccination, but have been comprehensively reviewed before (117–119), an innate lymphoid cell population, namely NK cells, has recently found its way into the spotlight of tuberculosis research.

## Natural Born Killers

Because of their ability to rapidly induce cell death without the need of further activation, for instance in response to virus infected cells and tumor cells, NK cells were classified as innate lymphoid cell population (120). However, upon infection, NK cells were found to display a memory-like phenotype providing so-called innate memory (121–123). This phenomenon has also been described upon *M. tuberculosis* infection (124). For instance, NK cells recovered from pleural fluid from tuberculosis patients were a major source of IFN-γ as well as IL-22 production when restimulated with *M. tuberculosis ex vivo* (125, 126). In murine tuberculosis models but also in patients latently infected with *M. tuberculosis*, NK cells expand, mature, and produce IFN-γ upon both, *M. tuberculosis* infection and BCG vaccination (127, 128). Venkatasubramanian et al. found reduced mycobacterial burden in the lungs of *M. tuberculosis*-infected C57 BL/6 mice after adoptive transfer of NK cells that matured and expanded in response to BCG vaccination. This observation is in contrast to findings by Junqueira-Kipnis et al. reporting similar lung CFUs of *M. tuberculosis*-infected C57 BL/6 mice after antibody-mediated depletion of NK cells, suggesting a priming effect of BCG, which induces a memory-like NK cell popuation. Thus, a potential protective role for NK cells during *M. tuberculosis* infection, especially in human individuals, requires further investigations (129). Indeed, a recent phase 1 clinical trial with 72 tuberculin skin test-positive participants from South Africa revealed that re-vaccination with BCG boosted frequencies of IFNγ-secreting NK cells in response to BCG (130). Notably, this was in contrast to BCG-specific CD4, CD8 as well as γδ T cells, which numbers were only transiently enhanced after re-vaccination. Sustained elevated frequencies of NK cells were still observed after 1 year.

The memory-like phenotype of NK cells in response to BCG is based on epigenetic reprogramming, a phenomenon also termed “trained immunity” (131) that has been observed also for myeloid-derived innate immune cells.

## Training Innate Immunity to Boost Vaccine Efficacy

The ability to develop an immunological memory has been attributed exclusively to lymphoid cells of the adaptive immune system, i.e., T and B cells. Recently, memory properties have also been described for prototypical cells of the innate immune system, namely macrophages and monocytes among others (132). Beneficial effects of BCG vaccination on several unrelated diseases, including non-mycobacterial infections, allergies, and cancer, in an antigen-independent manner have been comprehensively reviewed elsewhere with regard to innate memory characteristics (133–135). For instance, monocytes of BCG-vaccinated healthy individuals produced more IFN-γ, TNFα, and IL-1β in response to otherwise unrelated bacterial or fungal pathogens compared with the unvaccinated control group (136). The same study showed that 100% of severe combined immunodeficiency mice were protected from disseminated candidiasis after vaccination with BCG compared with only 30% of non-vaccinated mice. The authors conclude that BCG vaccination can instruct innate immune cells through epigenetic reprogramming based on histone methylation to provide antigen-independent protection in an NOD1 signaling pathway-dependent manner.

It remains to be elucidated whether targeting innate memory can be employed to improve anti-*M. tuberculosis* vaccination. As reviewed by Netea and van Crevel, several studies revealed that BCG-vaccinated household contacts, either juvenile or adults, of active tuberculosis patients were significantly less often interferon-gamma release assay (IGRA)-positive than non-vaccinated individuals, which was even more striking after a second BCG boost (133). The authors suggest that innate immune cells trained by BCG vaccination are able to eliminate *M. tuberculosis* before adaptive immunity kicks in leaving these individuals IGRA-negative, whereas those failing to clear the initial infection turn IGRA-positive. Corroborating these observations, Kagina et al. showed that house hold contacts of tuberculosis patients, which did not develop active tuberculosis, did not have increased mycobacteria-specific T cell responses (137). Thus, the capability of humans to control *M. tuberculosis* is not necessarily correlated with enhanced immune protection by antigen-specific T cells and, therefore, indicates the relevance of vaccination-triggered innate immune activation for clearance of an initial infection or, at least, reduction of the infection dose. Subsequent boost of acquired immune control may lead to latent tuberculosis without pathogen elimination. Identification of the vaccine associated triggers for innate immune cell training may thereby help to design better vaccine formulations to protect against active tuberculosis.

## Host-Directed Immune Modulation to Improve Vaccination Success

Concomitant administration of immune-modulating factors together with a vaccine has been studied mainly in other fields than tuberculosis. Especially in experimental cancer therapy, blockade of anti-inflammatory cytokines and T cell inhibitory receptors, such as TGF-β, programmed death-1, and CTLA4, respectively, resulted in increased T cell-mediated tumor regression (138, 139). Interestingly, administration of drugs that systemically suppress certain immune responses, namely the COX-2 inhibitors celecoxib and NS-398, enhanced tumor vaccine efficacies against breast cancer and pancreatic adenocarcinoma (140–142). Treatment of HIV patients with a COX inhibitor rescued T cell functions and humoral memory responses to tetanus toxoid, a T cell targeting vaccine (143). Of note, small lipid mediators produced by the cyclooxygenease pathway have been shown to attract neutrophils to the site of *M. tuberculosis* infection (13). Inhibition of neutrophil infiltration by Ibuprofen ameliorated tuberculosis pathogenesis and mycobacterial loads in susceptible C3HeB/FeB mice and, therefore, may be exploitable to improve anti-tuberculosis vaccines including BCG.

Macrophage responses may also serve as targets for host-directed therapies accompanying anti-tuberculosis vaccination. Taus et al. found that pretreatment of THP-1 cells with monosodium urate (MSU) crystals resulted in enhanced ROS production, promotion of phagosome–lysosome fusion, and increased killing of BCG by those cells, likely in an NOD-like receptor-mediated inflammasome-triggering process (144). MSU alone had no effect on BCG viability, indicating its pro-inflammatory properties, and capability to enhance anti-microbial responses. Moreover, BCG vaccination in the presence of MSU resulted in reduced colony-forming units in draining lymph nodes, showing increased killing of BCG *in vivo*. Again, BCG viability within the vaccine formulation was not affected by MSU. Interestingly, when mice were infected with *M. tuberculosis* Erdmann 10 weeks after vaccination, *M. tuberculosis* burden was reduced in the lungs and spleens, when the BCG vaccine was complemented with MSU in comparison with BCG alone. These findings raise the question, if enhanced killing of BCG upon vaccination, which is considered to counteract the maintenance of long-term immunity, may improve vaccine efficacy.

In another approach, the autophagy-inducing drug Rapamycin was used to successfully enhance processing and presentation of Ag85B by murine antigen-presenting cells (145). Immunization with Rapamycin treated dendritic cells led to increased Th1-mediated protection from *M. tuberculosis* infection. Thus, BCG vaccine efficacy may be enhanced by concomitant augmentation of autophagy.

Only a few studies assessed immune-modulatory approaches upon vaccination against tuberculosis. Surprisingly, repeated vaccine injections of BCG in already *M. tuberculosis*-infected BALB/c mice resulted in increased levels of IL-17, neutrophil influx into the lung, and tissue damage (146). This detrimental outcome of vaccination had been prevented by concomitant administration of a blocking anti-IL-17 antibody. Host-directed modulation of IL-17 upon vaccine treatment may have important implications for vaccinations in individuals that are already infected with *M. tuberculosis*.

**Figure 2:**
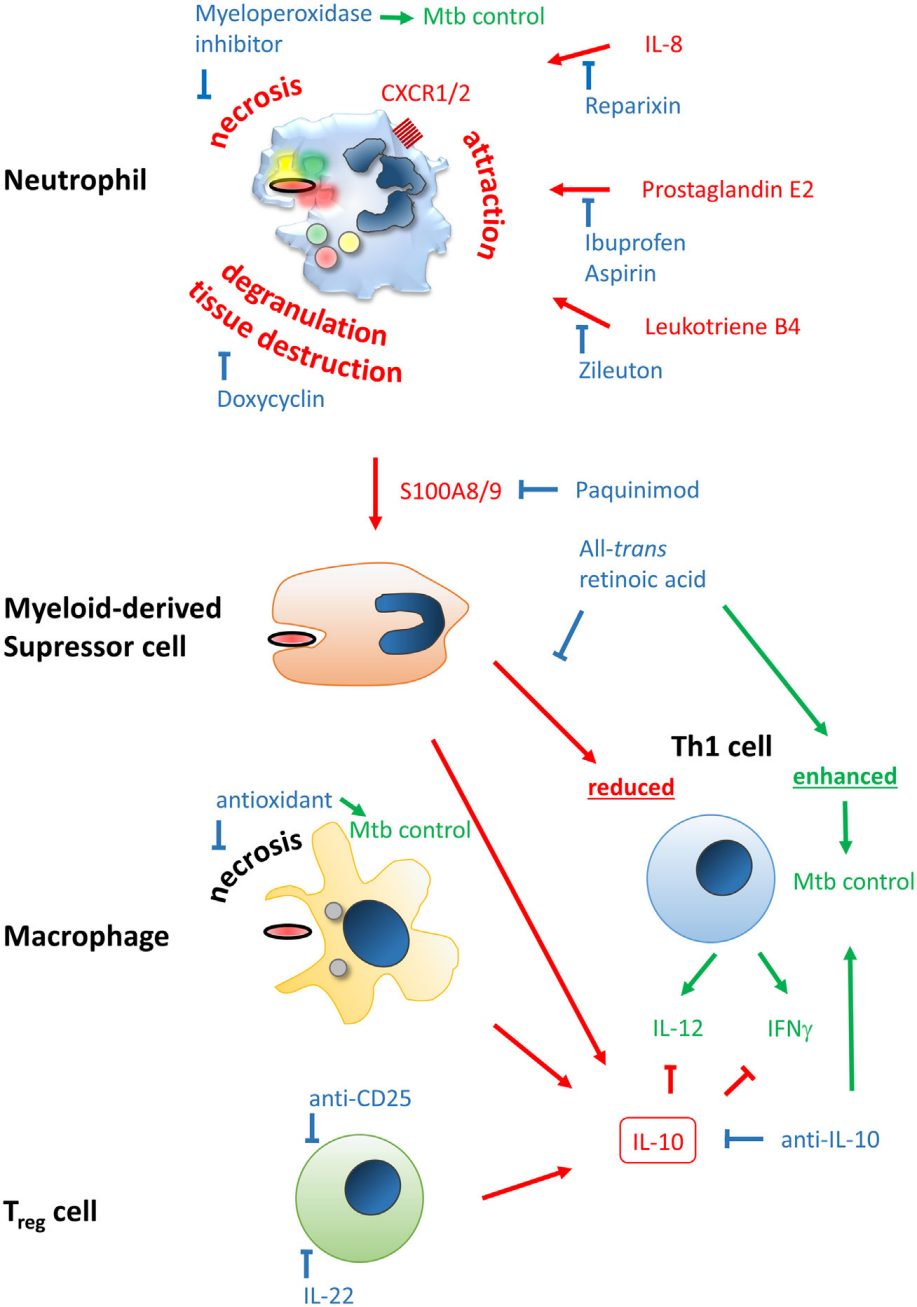
Improving efficacy of classical BCG vaccines by host-directed immune modulation. Potentially detrimental molecules, mechanisms, and outcomes leading to poor vaccine efficacy are color-coded in red, beneficial ones in green, and modulators thereof in blue. Neutrophil infiltration at the site of vaccination may limit vaccine efficacy and generation of a proper and lasting Th1 memory response. Neutrophil attraction is mediated by IL-8, prostaglandin E2, and leukotriene B4, which can be blocked by Reparixin, Zileuton, and Ibuprofen/Aspirin, respectively. The type of cell death upon vaccination may be beneficial or detrimental, i.e., immunogenic apoptosis vs. necrosis, respectively. The latter can be blocked by the myeloperoxidase inhibitor ABAH or anti-oxidants. Degranulation of neutrophils’ highly toxic molecules, preventable by Doxycyclin, may lead to tissue destruction and release of the alarmin S100A8/9 that attracts myeloid-derived suppressor cells (MDSC). S100A8/9 can be blocked by Paquinimod. MDSC inherit highly potent T cell-suppressive properties, potentially limiting establishment of Th1-mediated immunity. This mechanism can be counteracted by all-*trans* retinoic acid. Among MDSC, macrophages and regulatory T cells may be attracted to the site of vaccination. Together they shift the immune response to an IL-10-driven Th2 phenotype that inhibits protective Th1 responses shaped by IL-12 and IFNγ, which have been shown to be protective against *Mycobacterium tuberculosis* infection. Spatiotemporal treatment with a blocking anti-IL-10 antibody may rescue successful generation of a Th1 immune signature. Application of rIL-22 or anti-CD25 antibody may limit regulatory T cell (T_reg_) contribution.

Although there is only a small amount of data available, few publications show promising first results for increasing BCG vaccine efficacy by adjunct modulation of the host immune system. However, we can probably learn from data derived from tumor vaccination and other non-infectious disease vaccines boosted by concomitant immunotherapy. These results will be instrumental to develop novel strategies by temporarily adjusting the immune network to favor a long-term protective memory immune response against tuberculosis (Figure 2). Re-purposing already known host-directed therapeutics for anti-tuberculosis vaccination strategies may improve the protective efficacy, even of the “old” BCG.

## Conclusion

So far, novel strategies to boost vaccine efficacy against tuberculosis are based on enhancement of the BCG vaccine by addition of antigens that are expressed by *M. tuberculosis*, but not *M. bovis* BCG, e.g., ESX-1 secretion-mediating ESAT-6, TDM-synthesizing Ag85, and ESX-5-associated PE/PPE, or by genetically modifying BCG to express immunity-promoting mediators such as GM-CSF or IL-2 (147, 148). To improve vaccination efficacy against *M. tuberculosis* infection, focusing on more *M. tuberculosis* antigens may enhance the frequency of specific T cells, but may not solve the problem that BCG and next generation anti-TB vaccine types also elicit immunomodulatory functions that hamper immune-priming efficacy. Short-term shapeshift of the local immune responses upon vaccination may result in far-reaching long-term consequences for establishment of protective memory immunity against *M. tuberculosis* infection. These immune modulations may include regulation of neutrophil and MDSC influx to the site of vaccine application, prevention of detrimental kinds of cell death that interfere with generation of a protective memory immune response, and facilitation of other beneficial types of cell death, interference with T cell-inhibiting presentation of (self-)lipids after vaccination-mediated inflammation, temporal regulation of adverse, anti-inflammatory cytokine profiles, e.g., IL-10, and spatiotemporal modulation of immune cell subpopulations that suppress T effector cells, like T_regs_ and MDSC. Promising results of immune modulation during vaccination in other application areas, namely cancer vaccine research, highlight the potential of such host-directed improvements.

## Author Contributions

LL wrote the chapter “Lipid antigen antagonists to control vaccination efficacy?” EP wrote the chapter “Neutralizing anti-inflammatory IL-10 to improve vaccination success.” US and TD wrote all other parts of the manuscript and supervised the process. NR contributed Figure 1 and its legend. Additionally, TD contributed Figure 2 and its legend.

## Conflict of Interest Statement

The authors declare that the research was conducted in the absence of any commercial or financial relationships that could be construed as a potential conflict of interest.
